# Guitar Chord Sensing and Recognition Using Multi-Task Learning and Physical Data Augmentation with Robotics

**DOI:** 10.3390/s20216077

**Published:** 2020-10-26

**Authors:** Gerelmaa Byambatsogt, Lodoiravsal Choimaa, Gou Koutaki

**Affiliations:** 1 Department of Computer Science and Electrical Engineering, Kumamoto University, Kumamoto 860-8555, Japan; koutaki@cs.kumamoto-u.ac.jp; 2 Machine Intelligence Laboratory, National University of Mongolia, Ulaanbaatar 14201, Mongolia; lodoiravsal@num.edu.mn

**Keywords:** chord recognition, multi-task learning, guitar-playing robot, convolutional neural network, data augmentation

## Abstract

In recent years, many researchers have shown increasing interest in music information retrieval (MIR) applications, with automatic chord recognition being one of the popular tasks. Many studies have achieved/demonstrated considerable improvement using deep learning based models in automatic chord recognition problems. However, most of the existing models have focused on simple chord recognition, which classifies the root note with the major, minor, and seventh chords. Furthermore, in learning-based recognition, it is critical to collect high-quality and large amounts of training data to achieve the desired performance. In this paper, we present a multi-task learning (MTL) model for a guitar chord recognition task, where the model is trained using a relatively large-vocabulary guitar chord dataset. To solve data scarcity issues, a physical data augmentation method that directly records the chord dataset from a robotic performer is employed. Deep learning based MTL is proposed to improve the performance of automatic chord recognition with the proposed physical data augmentation dataset. The proposed MTL model is compared with four baseline models and its corresponding single-task learning model using two types of datasets, including a human dataset and a human combined with the augmented dataset. The proposed methods outperform the baseline models, and the results show that most scores of the proposed multi-task learning model are better than those of the corresponding single-task learning model. The experimental results demonstrate that physical data augmentation is an effective method for increasing the dataset size for guitar chord recognition tasks.

## 1. Introduction

Automatic chord recognition (ACR) is a fundamental problem in the music information retrieval (MIR) field, wherein a chord is one of the key elements for understanding music. ACR remains a challenging problem because of the richness of the acoustic signals, broad types, and complexity of the music signal. The purpose of chord recognition is the automatic recognition of the chord progression in music recordings and its labeling in an appropriate form, such as A:maj, A:min, and B:sus. Popular applications of ACR can be found in music identification, music segmentation, and similar music recommendation systems. Recent ACR studies have shown that the chord recognition performance can be significantly improved using deep learning models, especially convolutional neural networks (CNNs) and recurrent neural networks (RNNs) [1,2]. Many ACR studies have focused on simple chord recognition [3,4]. However, recent works have addressed the large-vocabulary chord recognition problem. One of the challenges of large-vocabulary chord recognition is that the distribution of chord classes in a dataset is highly unbalanced; some classes of chords appear less frequently than other classes in a dataset, thereby rendering it difficult to recognize the chords.

An ACR system consists of three main components, namely feature extraction, classifiers, and chord sequence decoding. In the feature extraction part, an input audio signal is converted to its time-frequency representation using a short-time Fourier transform or frequency spectrogram. The log mel-spectrogram and constant Q transform (CQT) are widely used features in chord recognition [4,5]. Moreover, many research works have employed chroma representation in feature extraction [6,7].

Deep neural networks (DNNs) and CNNs are widely used for classification in audio music classification tasks [1,8]. In recent years, multi-task learning (MTL) models have been successfully applied in many applications of machine learning. MTL can be observed in situations where knowledge is often transferred among tasks that are related. For example, in the case of human experience, the skills required for playing the guitar and ukulele can help improve the playing ability of each instrument. Similar to human learning, it is effective to learn multiple learning tasks simultaneously as the knowledge in a task can be utilized by other related tasks. MTL aims to learn more than one related task at a time [9] from a dataset and achieve better performance and learning efficiency in each task. It can also be used to solve data scarcity problems. We hypothesize that learning chord roots and qualities individually can improve the performance of the chord recognition model.

In this study, we used two types of datasets, namely the GuitarSet [10] and physical augmented dataset, which was first proposed in [11]. The GuitarSet dataset includes audio recordings from an acoustic guitar with various annotations, such as the chord label, tempo, and beat. The proposed physical augmented dataset includes audio recordings and only chord labels. Guitar-playing robotics was developed to create an augmented dataset. Robots have the advantage that they can be created with any design. Therefore, the robot musician has the benefit of playability that is associated with dynamic variation and playing speed.

In this paper, we address the problem of the relatively large-vocabulary chord recognition task by introducing multi-task deep learning architecture that learns the chord roots and qualities individually, and the output is integrated onto the symbolic label. Furthermore, we sought to generate an augmented dataset using robotics and prove its effectiveness via experimental evaluations. We also aimed to construct and open (https://github.com/gerelmaab/Physically_augmented_guitar_chord_dataset) a large-vocabulary dataset of guitar chords, which is created by the physical data augmentation method.5.

The rest of this paper is organized as follows. In Section 2, related works are introduced. Section 3 describes the musical chord recognition system including physical data augmentation, chord recognition, and the dataset. MTL and the implementation of its architecture are described in Section 4. In Section 5, the evaluation metrics and set of experiments performed are detailed. We compare our proposed methods, including multi-task learning and physical data augmentation methods, to the baseline models, equivalent single-task architecture, and human dataset, and we evaluate the results. Section 6 presents the conclusions with further directions.

## 2. Related Works

ACR has been studied in various forms over many years. One of the earliest works is that of Fujishima et al., titled “Realtime Chord Recognition of Musical Sound: a System using Common Lisp Music” [12]. In this work, the author proposed the calculation of a 12-D chroma feature that is compared to a dictionary of binary chord templates. Since then, ACR has gained popularity in MIR. In recent years, CNN-based models have outperformed the conventional methods. In [8], the authors were the first to evaluate the CNN performance in ACR tasks. In this work, the authors used a CNN to classify a five-second tile of pitch spectra, achieving a performance competitive to the standard techniques. Along with the CNN, RNNs have also been applied to MIR tasks. Boulanger-Lewandowski et al. studied the application of RNNs [13], wherein a RNN was used in the post-filtering phase for label sequence modeling. The RNNs were trained with the ground truth label sequences where the optimal label sequences were typically derived using the beam-search algorithm. McFee et al. [5] proposed large-vocabulary chord recognition based on a structured representation of the chord qualities. They used an extensive class of chord qualities, including triads, diminished, and suspended chords. A CNN and RNN can be used in one model, and this hybrid model is termed as a convolutional recurrent neural network (CRNN). Kenwoo et al. [14] showed that the CRNN benefits from the flexibility of the recurrent layers in summarizing information along time, thus achieving the best performance among the compared CNN structures in a music classification task.

A recent overview of MTL can be found in [15]. A MTL model based on a CNN architecture has achieved favorable results in computer vision tasks [16] and natural language processing tasks [17]. MTL has also been successfully employed in drug discovery [18] and chronic disease prediction tasks [19]. MTL has been applied to various MIR tasks owing to the various attributes of music, such as the chord, key, fundamental frequency, or baseline. The chord recognition accuracy can be increased with improved chord root recognition based on MTL [20]. In this work, the authors used cepstral features and a multi-task learning network based on neural networks to train chord recognition and root note recognition. In [21], the authors used a harmonic CQT feature and fully convolutional neural network based multi-task learning framework to estimate various tasks, including melody, vocal, and bass line estimation. The results showed that the proposed network outperformed the corresponding single-task network.

To date, different types of robot musicians have been developed [22]. Several studies have developed robotic guitars and basses, such as the LEMUR GuitarBot [23], and MechBass [24], a four-string robotic bass guitar. Another example is Strumbot [25], a standalone six-stringed robotic guitar. Besides these robotic guitars, robots that can play acoustic and electric guitars have also been developed.

## 3. Musical Chord Recognition System

In this section, we describe the proposed system and physical data generation method, and the input and output representations of the chord recognition system.

### 3.1. System Flow

**Figure 1:**
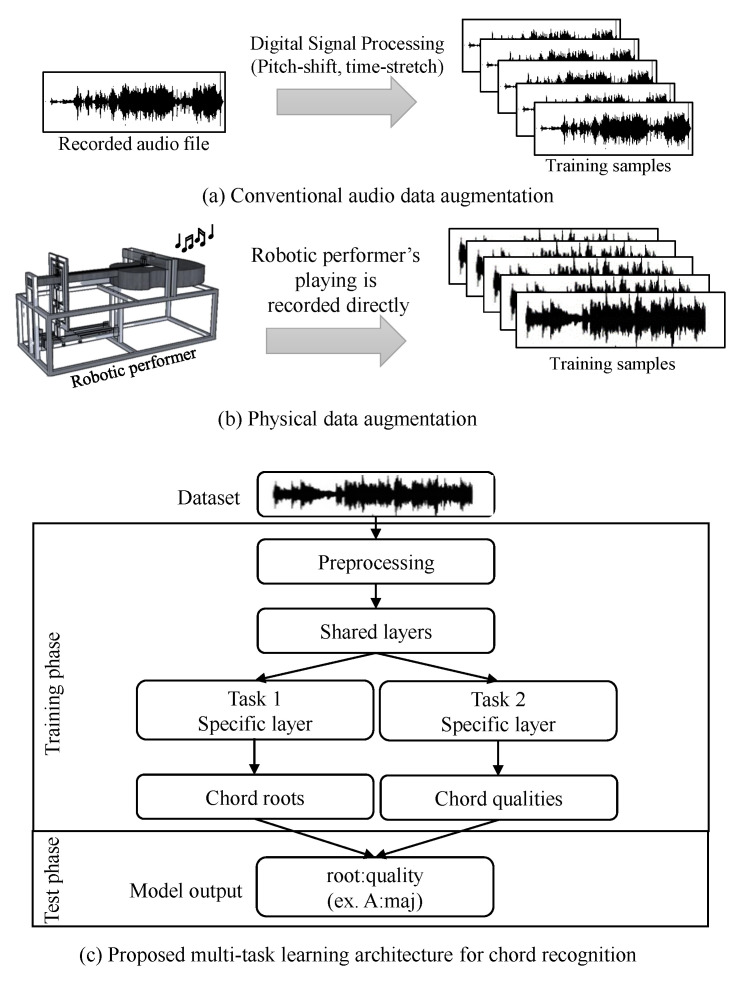
Proposed physical data augmentation method and multi-task learning method. (**a**) In the traditional method, digital signal processing techniques such as pitch-shifting or time-stretching are used to increase a small number of dataset samples. (**b**) In contrast, the proposed physical data augmentation method can generate high-quality training samples from the music performed by a controllable robot. (**c**) Dataset used to develop the proposed chord recognition model based on the multi-task learning (MTL) architecture. The proposed network architecture learns the chord root and qualities individually from the same audio file, and the model output is expressed using the ”root:quality” chord form.

The overall pipeline of the proposed recognition system is illustrated in Figure 1. In the data-driven approach, the quality and richness of the dataset are essential aspects. In this study, we used the physical data generation method to enrich and create a more balanced chord dataset, which is proposed in [26]. First, we developed a guitar-playing robot system that plays the chords automatically (Section 3.2). Then, a robotic performer was used to create the guitar chord dataset. A comparison between the traditional augmentation and physical data augmentation methods is shown in Figure 1a.

The proposed solution for the recognition system involves a multi-task learning model based on a CNN and RNN. The MTL model predicts two tasks in parallel, as illustrated in Figure 1b. The input of the recognition system is derived from the physical augmented dataset. The proposed system uses two-dimensional (2D) representations instead of the original one-dimensional (1D) representation of audio. The audio signals are first pre-processed and decomposed into small frames, and frame-wise feature extraction is performed. We tailored the feature extraction strategies to produce suitable inputs for the network (Section 3.3.2).

In the training phase, two types of related tasks are trained simultaneously. The two tasks take the same single input. The given input audio has multiple labels (root note and quality). The output root note and quality, respectively, are estimated/generated and then combined into one label. The output of the learning models is converted into label files to evaluate the performance of the architectures. We tested the recognition system only on a human dataset.

### 3.2. Physical Data Augmentation Guitar Playing Robot

**Figure 2:**
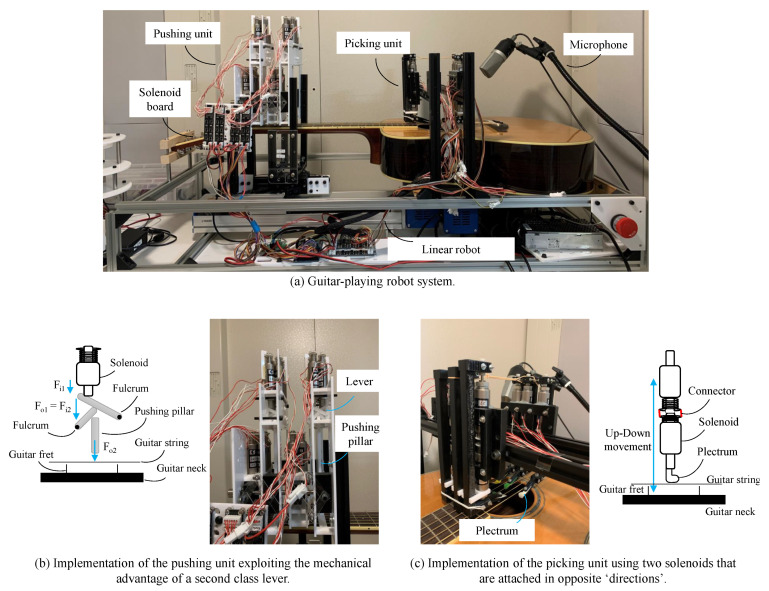
The developed guitar-playing robot system. The robot system consists of a single-axis robot, string-pushing unit, picking unit, and an actual guitar. (**a**) The guitar-playing robot system. The microphone is placed near the guitar sound hole. (**b**) Implementation of the pushing unit. The mechanical advantage of a second class lever is exploited. A rubber band is used to move the pushing pillar back to its original position. (**c**) Development of the picking unit. Two solenoids are placed in opposite directions. Therefore, the plectrum can be moved along two directions (up and down).

The robot system comprises a string-pushing unit, picking unit, and linearly moving mechanical unit. A snapshot of the developed guitar-playing robot system is shown in Figure 2. The chassis of the robot was built with a T-slot aluminum extrusion, and the other supporting parts were made with acrylic and polylactic acid (PLA) filament. This type of robotic performer was also implemented in [26].

#### 3.2.1. Pushing Unit

**Figure 3:**
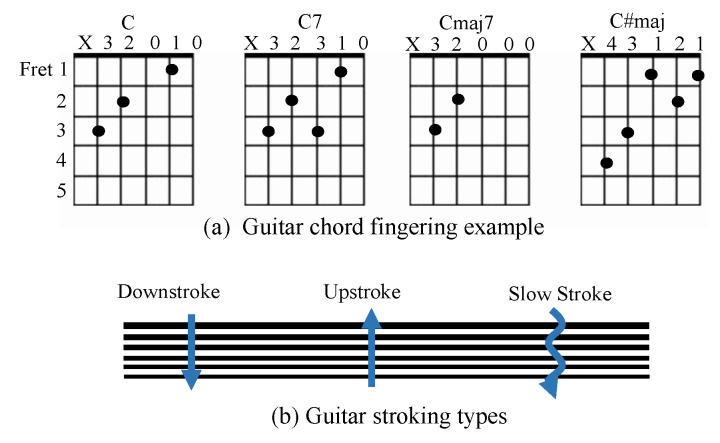
(**a**) Guitar fingering of the C chords. There are many forms of chords denoted by the same chord name ‘C’. The robot guitarist plays exhaustive combinations of different chord forms and strokes automatically, and the sounds are recorded as training samples. (**b**) Types of strokes.

The guitar-playing robot pushes the strings correctly at the right positions and subsequently generates various types of tones from the guitar. For the string-pushing unit, a four-line solenoid bar with six solenoids used in each line to fit six strings were installed; in total, there are 24 solenoids, so that any variation of a chord can be played as shown in Figure 3. The solenoid bars are moved along the guitar neck, assisted by the single-axis robot. Figure 2b illustrates the implementation of the pushing unit, whose main body is made of acrylic. To produce at least 60 N from the push–pull solenoid, which is the required preload tension to generate an accurate tone, we used the double second class lever in which the mechanical advantage (MA) is always greater than 1 (Figure 2b). The law of levers is defined by Equation (1).

(1) $$\begin{array}{c} MA=\frac{F_{o}}{F_{i}}=\frac{d_{i}}{d_{o}} \end{array}$$

Hence, the input force (F${}_{i1}$) is approximately three times the output force (F${}_{o2}$). When sufficient force is applied to the pushing pillar, it pushes the string. A rubber band is used to move the pushing pillar up to its initial position.

#### 3.2.2. Picking Unit

The robot can pluck the string by a linear or swinging motion near the guitar sound hole to generate sound. Normally, a human would make a vertical movement to pluck a guitar string; however, instead of a vertical movement, our robot makes horizontal a movement, as illustrated in Figure 2c. The picking unit was implemented with push–pull solenoids similar to the pushing unit. Each solenoid of the picking unit executes a controllable linear up and down motion using two solenoids that are deployed in opposite directions to each other. Six plectrums are attached to the picking unit, with one plectrum per string, and the guitar pick is designed to fit between two strings, as shown in Figure 2c. The plectrum holder was constructed using a 3D printer with a PLA filament. The picking units were installed above the guitar sound hole in a fixed position. Each picking component is independently controllable; therefore, the apparatus can be strummed using many types of strokes. The proposed robot system can play various strokes, including the fast, slow, up, and down strokes, as illustrated in Figure 3b.

#### 3.2.3. Linear Movement

Common guitar chords can generally be played below the 5th fret. The pushing unit moves along with the guitar neck, to facilitate a variety of chords. The solenoid carriage, attached to a linear moving robot, moves across the first 12 frets of a guitar. The solenoid carriage rides along the T-slot aluminum extrusion, as shown in Figure 2a.

#### 3.2.4. Electronic Controller

Electronics were used to control all units including the controlling board, stepper motor drivers, solenoid driver circuits, and power supply, which is used to power both the motors and electronics. We constructed an electronic board to control the solenoids, including six solenoid controlling circuits on one board, as shown in Figure 2a. The solenoids operate at 12 V and are controlled by an on/off voltage. Four boards are used to control the 24 solenoids. The controlling board was connected to the computer via a USB. The message from the computer to the controlling board contains chord information. Upon receipt of a message, the controlling board communicates with the stepper motor driver, driving the linear mechanism to a position corresponding to the received chord or note position. Subsequently, the corresponding solenoids are activated, and the accurate chord is played.

### 3.3. Musical Chord Recognition Part

We designed a multi-task architecture that jointly learns root note recognition and chord quality recognition in the training phase. In musical chord recognition, feature extraction operates over the frames. The pre-processed input is passed through two identical CRNNs to generate the root as the first output and quality as the second output. Each output of the sub-networks is then combined to obtain a single simple chord annotation form.

#### 3.3.1. Input Representation

Many research works have shown that the time versus frequency representation is most suited for learning-based approaches in music [2,14]. We used the CQT as an input representation of the models. The CQT is a logarithmic representation of frequency, which can be easily calculated from a raw audio [27]. The main advantage of the CQT representation is that higher-frequency resolution is obtained at lower frequencies and higher time resolution is achievable at higher frequencies.

**Figure 4:**
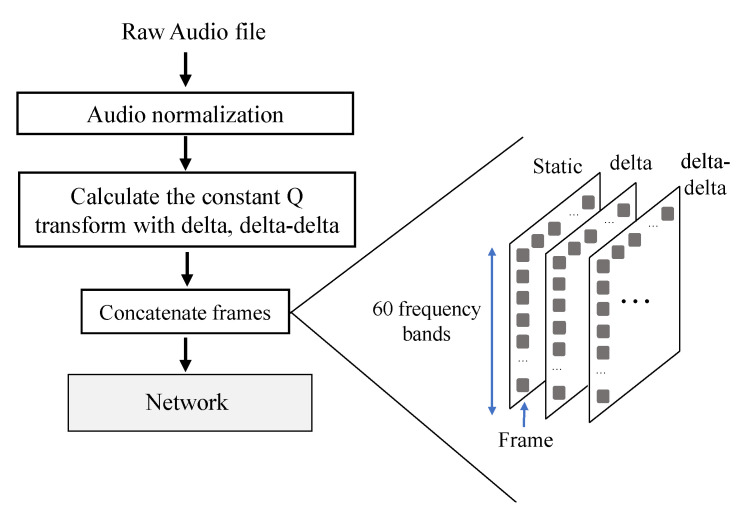
Block diagram showing the audio pre-processing steps. First, the audio is normalized using basic audio normalization, and the constant Q transform (CQT) feature along with the delta and delta-delta features are subsequently computed.

Abdel-Hamid et al. used the Mel-frequency spectral coefficients (MFSC) feature for speech recognition tasks along with their delta and delta-delta to describe the acoustic energy distribution in each of the several different frequency bands [28]. This representation is similar to the red, green, and blue channels of an image. These dynamic (delta) and acceleration (delta-delta) features are very efficient in speech recognition tasks [29]. Inspired by the above, the CQT, along with the corresponding delta and delta-delta features (first and second temporal derivatives of the CQT), can be applied to the chord recognition task. We can assume that the delta and delta-delta features can achieve improved performance at the chord boundaries, as the delta features have large values with sound changes. We experimentally demonstrate the effectiveness of the delta and delta-delta features. (Section 5.1) The block diagram depicting all the pre-processing steps is illustrated in Figure 4.

Librosa [30] computed the CQT feature with a 4096-point hop size, resulting in a frame rate of approximately 10.8 Hz. In an acoustic guitar, the lowest note is E2, which has a fundamental frequency of 82 Hz, and the highest note is F6, which has a fundamental frequency of 1397 Hz. Therefore, we used the logarithmic CQT parameters to span five octaves, starting at the A1 note at about 55 Hz with 12 bins per octave. The preprocessing parameters are listed in Table 1. The output dimensions of this processing step were one frame × 60 bins.

The neighboring frames of the input representation can be expected to contain similar content, as the chords will not change on a frame-by-frame basis. Through systematic experiments on the validation folds (Section 5.2), we found that the scores of the root and quality are stabilized from a context window of ±0.45 s. In this study, each time 11 consecutive frames are input to the proposed model, frames around the target frame ±5 frames (±0.45 s audio clip) are chosen, which is termed as a superframe. The superframe is an approximately 1.0 s audio clip. This operation is applied to all the frames. The output of the pre-processing step is a 3 × 11 × 60 sized array, where 60 represents the number of CQT bins, 11 is the total number of frames, and 3 represents the CQT feature along with its derivatives.

#### 3.3.2. Output Representation

In the dataset annotation, chord description is represented as G#:maj6(2,b5,5)/1. To formulate chord recognition as a classification task, we define a mapping of the dataset from chord description to chord vocabulary. First, we discard the chord inversions and suppressed or additional notes. For example:

*D#:sus2(7)/1 → D#:sus2*

Next, we introduce some conversion from the chord label with a short duration to the analogy label. We use ten types of quality, including maj, min, aug, maj7, min7, 7, dim7, hdim7, sus2, and sus4. The final vocabulary contains 98 classes. We incorporate a conversion over a chord as follows:
maj6 → majmin6 → minmaj9, minmaj7 → maj7min9, min11 → min79, 11 → 7dim → dim7

**Figure 5:**
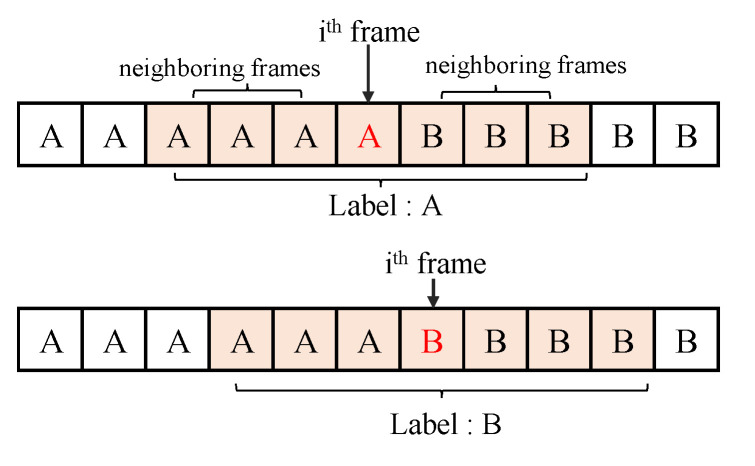
Example of labeling an audio context clip. (**Top**) ith frame corresponds to A chord, and the label of this audio context is A chord. (**Bottom**) ith frame corresponds to B chord, and the label of this audio context is B chord.

For the labeling, a single label is estimated with the superframe. The chord label of the middle frame is used to the audio context clip label as illustrated in Figure 5.

### 3.4. Datasets

*The GuitarSet dataset*: The GuitarSet contains musical audio files and the corresponding chord annotations, which were annotated with [31]. Three chord progressions are paired with five different genres, including rock, jazz, singer-songwriter, funk, and bossa nova. Six professional guitarists played an audio of 30 min audio in their own style, to produce 3 h of 180 audio recordings. There are two different chord annotations termed as the “instructed” and “performed” chord annotations. The “instructed” chord annotation is written with the given chord sheet. In contrast, guitarists have modified the given chords to their playing style, which is called the “performed” annotation. These two types of annotations are not necessarily the same; however, the audio file is the same. As instructed chord label is modified with different chords by the players, we used the “performed” chord annotation.

*The augmented dataset*: The augmented dataset was recorded directly from the guitar playing robot. This dataset consists of 12 root notes and 10 types of chord quality in a total of 97 classes of chords. Each chord was played individually with five types of stroking patterns, including D DU DU DU D, D DU UDU, D D DU UDD, D D DU, and simple fingerstyle, where D represents a downstroke, U represents an upstroke, and a space represents the gap time between the strokes. The recording environment was a sound isolation chamber. The microphone was placed near the guitar sound hole. Each specific chord was recorded for approximately 40–45 s, indicating that the created augmented dataset was evenly distributed over all the chord types. This dataset was recorded in an identical manner as the human dataset, in the WAV format and sampled at fs = 44,100 Hz. We annotated the chords manually using the chord annotation method proposed by [31].

**Figure 6:**
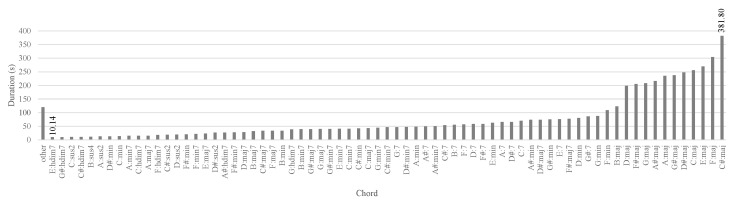
Distribution graph of the chord types in the Guitarset dataset. Here, “other” includes 27 classes of the chord with a duration of 1–9 s.

The Guitarset dataset is an unbalanced dataset over the chords, e.g., the C#:aug and C#:sus4 chords have the least duration of approximately 1.2 s; in contrast, the C#:maj chord has the longest duration of approximately 380 s. The distribution of the chord types of the Guitarset dataset is shown in Figure 6. To address this data scarcity or unbalanced problem, we propose a multi-task learning network. The proposed multi-task learning network trains the chord root and quality separately. Observing the chord distribution based on the chord root and quality in Table 2 and Table 3, respectively, it can be seen that the model has considerably more information on the chord in terms of root and quality. It can be observed from the tables of the roots and qualities that the training examples are increased compared to all the chord classes. However, the chord qualities are also not flat (unbalanced); dim7 accounts for approximately 0.07% of the chord quality distribution, which is considerably smaller than that of the major chords, which is approximately 52.58%. In contrast, the root note is relatively evenly distributed over the GuitarSet dataset. In an augmented dataset, all chord types are at least approximately 45 s long.

## 4. Proposed Multi-Task Learning

This section details single-task learning and the structure of the proposed MTL model.

### 4.1. Conventional Single-Task Learning

**Figure 7:**
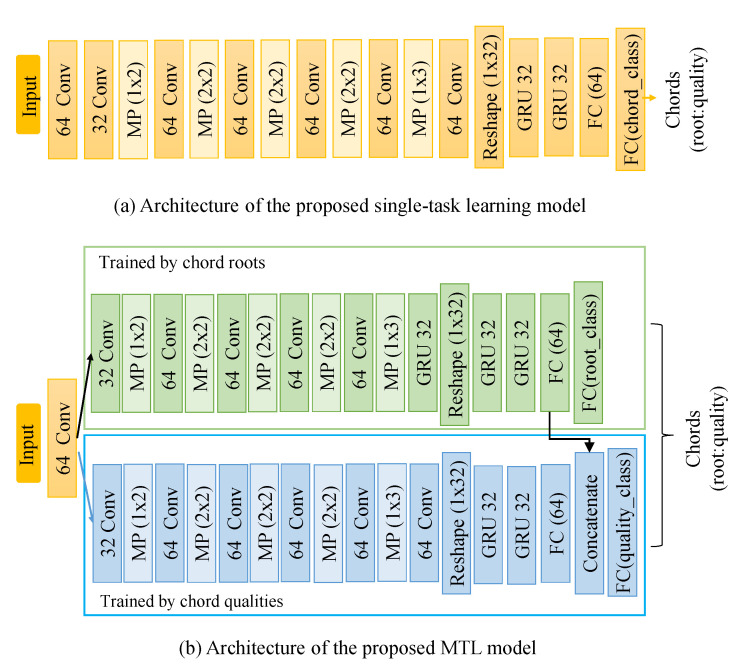
Architectures: (**a**) Single-task learning architecture that is identical to the sub-network of the multi-task learning architecture. (**b**) Multi-task learning model. The chord root and qualities are trained by specific networks from the same dataset, and the output is presented in the form of root:quality. Here, MP represents the max-pooling layer, and FC is a fully connected layer.

The single-task learning network contains seven convolutional layers, two recurrent layers, and two fully connected (FC) layers. The architecture of the proposed convolutional recurrent neural network (CRNN) is shown in Figure 7a. The proposed architecture contains seven convolutional layers with a 3 × 3 receptive field and a rectified linear unit (ReLU) activation function, followed by batch normalization [32]. Five max-pooling layers of various sizes are employed, followed by a dropout [33] with 0.5 probability. We reshaped the output of the convolutional layer to make it compatible with the input size of the recurrent layer. A gated recurrent unit (GRU) with 32 neurons was used as the recurrent layer. The output of the recurrent layer was connected to the FC layer with 64 neurons, and the ”softmax” activation function was used for output classification. The single-task learning model is used for comparison in the subsequent experiments.

Training deep learning systems usually requires large and balanced datasets to train a good system and learn accurate parameters. However, in some applications such as medical imaging or where there is a relative lack of data (certain classes of datasets are rare relative to other events, objects, or classes) this requirement cannot be satisfied. In a large-vocabulary chord dataset, the majority class examples, such as major and minor, far outnumber the minority class examples such as suspended or augmented. In these cases, the single-task learning model lacks knowledge of some classes, and accurate learning is difficult. However, multi-task learning is a useful approach in cases where useful information can be derived from other related learning tasks to handle the data sparsity problem.

### 4.2. Multi-Task Learning

The proposed model learns a mapping between an audio representation, such as the CQT, and the root notes and qualities. Essentially, the model can be viewed as a multi-task leaning model. From the pre-processed input, the proposed model computes the root and quality outputs directly. The model is trained with a multi-task learning method using two cost functions. In each cost function, the cross entropy (CE) between the ground truth and predicted labels is used. In summary, the root note and quality are modeled jointly with a shared network by the proposed model. This learning method is designed for chord recognition.

In the case where the labeled data for one of the tasks are scarce, MTL can be adapted to the labeled data of the related task. In our dataset, the suspended, augmented quality training examples are short compared with the other classes of quality. As the two tasks have a different number of outputs, the potentially shareable layers are the middle layers of the model, resulting in a final layer of different dimensionality. In the proposed model, one sub-network learns 12 root notes, while the other sub-network learns 10 types of qualities, including major, minor, dominant seventh, dominant major and minor seventh, suspended, augmented, and half diminished 7 and diminished 7. The outputs of the two networks are then combined to obtain the ”root:quality” annotation form, which was proposed in [31]. In this approach, the number of output classes reduces to 12 and 10 for the root and quality, respectively, compared with the single-task learning model, with no change in the number of training examples.

### 4.3. Multi-Task Learning Architecture

We combined MTL with the CNN and RNN framework by sharing some layers between the two related tasks. The input features are passed through two identical sub-networks, where each sub-network architecture is the same as a single-task learning model except for the output layer. The two identical architectures perform root note and quality recognition. The output layers have a different number of outputs. The output of the first task is 12 because there are twelve music notes. The output of the second task is 10 because ten types of chord qualities are employed. The proposed MTL model is shown in Figure 7b.

The MTL model has one shared layer and uses soft parameter sharing in the FC layers of the two sub-networks. The output of the FC layer of the root learning sub-network is concatenated to the output of the FC layer of the quality learning sub-network. Therefore, the classification of quality has some information of the chord root.

### 4.4. Training

In the experiment, 6-fold cross-validation was applied to the entire dataset. In each training fold, five players’ recordings were used for training, while the other was used for testing the dataset. First, the complete network was trained only with the human dataset (Guitarset dataset). Subsequently, the physical data augmented dataset was combined with the training subset of the human dataset, and the same experiment as the first was performed. In the second experiment, the test subset only consisted of the human played chords, similar to the first experiment.

In the training phase, a frame-based strategy was used, wherein each song was divided into frames, and each frame was treated as an independent training sample. On average, each song was divided into approximately 250–350 frames, resulting in approximately 32,500 and 75,000 training samples in the human and augmented datasets, respectively, and approximately 9800 test samples.

The neighboring frames of the input representation can be expected to contain similar content, as the chords will not change on a frame-by-frame basis. Therefore, the models were trained with 11 frames (of 1 s duration). The networks were implemented using the Keras library that runs on top of TensorFlow. The Adam optimizer [34] was employed to train the networks for up to 300 epochs with a learning rate of 0.001 and a batch size of 100. Training was stopped early if there was no improvement in the validation loss after 10 epochs.

## 5. Experimental Result

Weighted chord symbol recall (WCSR) was used for evaluation. The WCSR score can be computed using Equation (2), where *t_c_* is the duration of the correctly classified chord segments and *t_a_* is the duration of the entire chord segments.

(2) $$\begin{array}{c} WCSR=\frac{t_{c}}{t_{a}}*100\% \end{array}$$

The WCSR score was computed with mir_eval [35]. The root, maj-min, thirds, triads, sevenths, tetrads, and MIREX scores were used to compare the results. In mir_eval, the root only compares chord root; maj-min compares the 12 major, 12 minor, and ’no chord’ classes; thirds compare root and thirds; triads compare root, third, and fifth; sevenths compare root, thirds, fifths, and sevenths; tetrads compare all intervals, and MIREX compares at least three correct notes. The output of the learning models was converted into label files to calculate the scores of mir_eval.

### 5.1. Effects of CQT with Delta, Delta-Delta Features

In this experiment, cross-validation was not employed. In both the model cases, the GuitarSet dataset was used to train and test the models. Five players’ recordings were used as the training dataset, and the other player’s recordings were used as the test dataset. The CNN model is a VGG-style [36] CNN. Table 4 shows the advantages of using the delta and delta-delta features, resulting in significantly improved accuracy in both the models and datasets.

### 5.2. Effects of Audio Context Size

**Figure 8:**
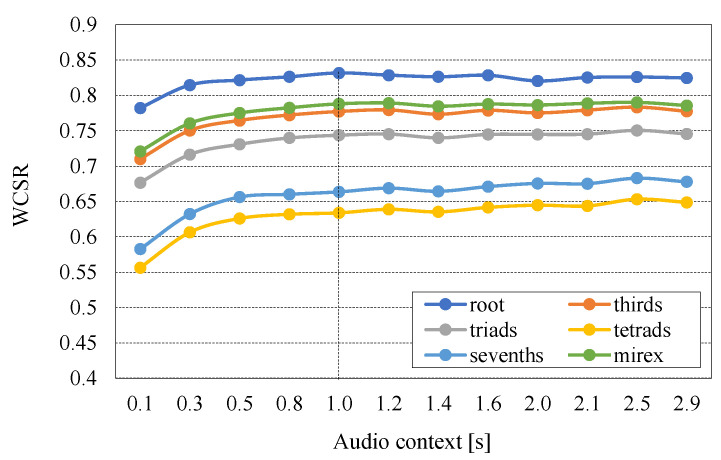
Validation WCSR for chord recognition of different scores based on different audio context sizes in the VGG-style CNN network.

Here, a VGG-style CNN architecture was used to evaluate the context size. We determined the optimal amount of context for each score experimentally using the one-fold experiment, as shown in Figure 8. The results indicate that the scores were nearly stable from 1 s onwards, which has a context size of 5. Therefore, we chose a 1 s audio context for all the experiments.

### 5.3. Effects of Traditional Augmentation and Physical Augmentation Methods

In this experiment, we used the simple CNN model with the CQT and delta and delta-delta features, along with a small dataset. In the data augmentation method, time-stretching data augmentation was used, which slows down or speeds up the audio sample while keeping the pitch unchanged. Each sample was time-stretched using four randomly selected factors: 0.8, 0.9, 1.1, and 1.2. Four types of metrics were used for comparison because a small dataset was employed. Table 5 shows the advantages of using the physical data augmentation method, which results in improved accuracy.

### 5.4. Overall Performance

Here, we compare the performance of the proposed and baseline methods on the same datasets. It is not simple to define a particular method as a state-of-the-art method because the chord recognition methods were evaluated on different datasets. Among the different models, we chose four baseline methods, namely the deep neural network (DNN) model, VGG-style CNN model, CNN [4] model, and combination of a CNN and gated recurrent unit, termed CR2 in [5]. The DNN has two hidden layers with 128 and 64 neurons, and an output layer. The softmax activation function is used for classification. In the VGG-style CNN architecture, four convolutional layers with a 3 × 3 filter are followed by a max-pooling layer of size 2 × 1. After this, two convolutional layers and max-pooling layers of size 2 × 2 are connected. This is followed by one convolutional layer and max-pooling layer of size 1 × 2. A FC layer with a softmax activation function is used for classification. Dropout is applied after each max-pooling and FC layer. The CNN [4] includes eight convolutional layers, two max-pooling layers, and an average pooling layer instead of a flatten layer. Dropout and batch normalization [32] are used to prevent overfitting and speed up training convergence, respectively. The CR2 model [5] consists of two convolutional layers and two bidirectional GRU units. The baseline models were re-implemented to suit our input feature representation.

The experimental results of the baseline models and proposed methods are presented in Table 6. The best value obtained for each metric is highlighted in bold. Among the baseline models, the VGG-CNN model, which is baseline model 2, achieved the best results in terms of most metrics. When the augmented dataset was used, the quality scores increased except in the case of the CR2 model. When models with single-task learning were included, the best performance was obtained in terms of all the metrics.

When comparing the proposed methods, the MTL model trained with the augmented dataset, which was proposed method 3, obtained better performance in terms of four metrics, namely the root, thirds, tetrads, and maj-min. The other metrics showed comparable performance with the proposed methods. The STL performed better for the triad, sevenths, and MIREX. The proposed method achieved better performance than the baseline models and single-task learning model. The MTL models achieved better scores for chord root and triad recognition.

### 5.5. Discussion

Based on the above results, a CNN proved more effective for the chord recognition task compared with the traditional DNN learning method. The proposed MTL model based on the CNN and RNN networks showed good performance for the large-vocabulary guitar chord recognition task. The presented results clearly demonstrate that the performance of the proposed method was superior to that of the baseline models and single-task learning model. We demonstrated that the static feature along with the dynamic (delta) and acceleration (delta-delta) features are useful for the guitar chord recognition task. In contrast, the physical data augmentation method showed acceptable performance in the guitar chord recognition task. This physical data augmentation method can be used for any musical instrument. Using a robotic performer, we can create a large-sized and balanced dataset, which also includes sufficient training examples of the rarely played chords such as the sustained and eleventh. However, there are some limitations, including the hardware implementation and complexity of the robot design.

## 6. Conclusions

In this paper, we applied a multi-task learning model based on a CNN and RNN to a relatively large-vocabulary guitar chord recognition task and demonstrated the physical data augmentation method and its utility/performance. The experimental results demonstrated that the multi-task learning model outperformed the baseline models and achieved better performance than its equivalent single-task model. In addition, we found that an augmented dataset created by a robot is also efficient for guitar chord recognition. Both dynamic and acceleration features were used as the model’s input features, resulting in good performance. The models trained by a human with the augmented dataset achieved improved performance in terms of most of the metrics. An advantage of multi-task learning for chord recognition is that its training speed is faster than that of single-task learning.

## Figures and Tables

**Table 1:** Preprocessing Parameters.

Parameters	Value
Time frame	0.092 s
Sampling rate	44,100
FFT frame size	8192
Hop size	4096
Starting Frequency	55 Hz
Bins per octave	12
Number of octaves	5

**Table 2:** Root Note Distribution.

**Chord Root Note**	B	D	F#	A	A#	C	G	D#	E	G#	F	C#
**Percent** (%)	5.47	7.11	7.43	7.46	7.86	8.42	8.56	8.69	8.98	9.13	10.11	10.79

**Table 3:** Chord Quality Distribution.

**Chord Quality**	dim7	aug	sus4	sus2	hdim7	min7	maj7	min	7	maj
**Percent** (%)	0.07	0.29	1.08	2.19	2.51	7.63	8.11	12.02	13.53	52.58

**Table 4:** Weighted Chord Symbol Recall (WCSR) Scores of the Compared Methods. The Best Values are Represented in Bold. (Δ represents delta and delta-delta).

Model	Feature type	Root	Thirds	Triads	Sevenths	Tetrads	Maj-Min	MIREX
CNN ${}^{1}$	CQT ${}^{2}$	0.822	0.768	0.736	0.658	0.631	0.771	0.780
	CQT with Δ	**0.832**	**0.777**	**0.744**	**0.664**	**0.634**	**0.782**	**0.788**
CRNN ${}^{3}$	CQT	0.730	0.655	0.623	0.544	0.517	0.659	0.676
	CQT with Δ	**0.754**	**0.682**	**0.651**	**0.571**	**0.545**	**0.685**	**0.699**

${}^{1}$ Convolutional neural network. ${}^{2}$ Constant Q transform. ${}^{3}$ Convolutional recurrent neural network.

**Table 5:** WCSR Scores of the Compared Methods. The best Values are Represented in Bold. (DA Represents Data Augmentation).

Model	Root	Maj-Min	Sevenths	MIREX
(a) no DA	0.794	0.724	0.665	0.756
(b) (a) + DA	0.831	0.783	0.736	0.792
(c) (b) + physical DA	**0.862**	**0.815**	**0.771**	**0.821**

**Table 6:** Median-Weighted Recall Scores of the Compared Methods. The Best Values are Represented in Bold. (aug. refers to the augmented dataset).

Experiment	Root	Thirds	Triads	Sevenths	Tetrads	Maj-Min	MIREX
DNN (Baseline 1)	0.772	0.657	0.618	0.497	0.474	0.644	0.672
DNN with aug. (Baseline 1)	0.770	0.656	0.625	0.518	0.493	0.649	0.669
VGG-CNN (Baseline 2)	0.842	0.798	0.772	0.686	0.658	0.805	0.82
VGG-CNN with aug. (Baseline 2)	0.842	0.792	0.766	0.692	0.665	0.800	0.823
CNN(Baseline 3)	0.797	0.733	0.701	0.620	0.596	0.732	0.744
CNN with aug. (Baseline 3)	0.805	0.732	0.692	0.626	0.600	0.721	0.742
CR2 (Baseline 4)	0.791	0.697	0.664	0.560	0.538	0.693	0.721
CR2 with aug. (Baseline 4)	0.779	0.685	0.656	0.555	0.535	0.679	0.711
STL model (prev)	0.861	0.816	**0.793**	**0.708**	0.678	0.824	**0.836**
STL with aug.(prop1)	0.870	0.814	0.786	0.706	0.677	0.817	0.823
MTL model (prop2)	0.880	0.817	0.781	0.703	0.675	0.815	0.812
MTL with aug. (prop3)	**0.882**	**0.826**	0.792	0.705	**0.697**	**0.829**	0.820

## Abbreviations

The following abbreviations are used in this manuscript:

MIR	Music Information Retrieval
MTL	Multi-Task Learning
ACR	Automatic Chord Recongition
CNN	Convolutional Neural Network
RNN	Recurrent Neural Network
MFSC	Mel-Frequency Spectral Coefficients
MP	Max-Pooling
FC	Fully Connected
STL	Single-Task Learning
GRU	Gated Recurrent Unit
ReLU	Rectified Linear Unit
CQT	Constant Q Transform
MA	Mechanical Advantage
LR	Logistic Regression
MLP	Multi Layer Perceptron
DNN	Deep Neural Network
WCSR	Weighted Chord Symbol Recall
